# Prevalence of Anemia and Iron Deficiency among Palestinian Pregnant Women and Its Association with Pregnancy Outcome

**DOI:** 10.1155/2018/9135625

**Published:** 2018-12-24

**Authors:** Mahmoud A. Srour, Samah S. Aqel, Khaled M. Srour, Khalid R. Younis, Fekri Samarah

**Affiliations:** ^1^Department of Medical Laboratory Sciences, Faculty of Health Professions, Al-Quds University, East Jerusalem, State of Palestine; ^2^Department of Biology & Biochemistry, Birzeit University, Birzeit, State of Palestine; ^3^Anatomy and Physiology Department, Faculty of Medicine, Al-Quds University, East Jerusalem, State of Palestine; ^4^Department of Medical Laboratory Sciences, Arab-American University, Jenin, State of Palestine

## Abstract

**Background:**

Anemia is a public health problem especially among pregnant women. This study aimed to investigate the prevalence of anemia and iron deficiency among pregnant women and its association with pregnancy outcome in Hebron Governorate in southern Palestine.

**Methods:**

This is a cross-sectional study that included 300 pregnant women in their first trimester and 163 babies. Maternal anthropometric and socioeconomic and newborns' data were collected. Complete blood count for study subjects and maternal serum ferritin were measured.

**Results:**

The prevalence of iron deficiency anemia among pregnant women was 25.7% and 52% of them had depleted iron stores. When pregnant women were grouped into three hemoglobin (Hb) tertile groups, a significant difference was observed between maternal Hb and newborns' birth weight (*P*= 0.009), height (*P*= 0.022), head circumference (*P*= 0.017), and gestational age (*P*= 0.012). There was a significant association between maternal serum ferritin and frequency of low birth weight (*P*= 0.001) and frequency of preterm delivery (*P*= 0.003). No significant association was observed between maternal anthropometric measures or the socioeconomic status and pregnancy outcomes.

**Conclusion:**

Iron deficiency is a moderate public health problem among the study subjects. Maternal Hb and serum ferritin significantly affect pregnancy outcomes.

## 1. Introduction 

Anemia is a widespread public health problem associated with an increased risk of morbidity and mortality, especially in pregnant women and young children. Anemia also negatively influences the social and economic development in countries with high prevalence of anemia [1, 2]. In general, it is assumed that 50% of the cases of anemia are due to iron deficiency [3, 4]. Iron deficiency anemia (IDA) is considered to be one of the top ten contributors to the global burden of disease [1, 2].

Iron deficiency is the most common nutritional deficiency worldwide, particularly among pregnant women. Because of the increased iron requirements during pregnancy, pregnant women are recognized as the group most vulnerable to iron deficiency anemia [2, 5]. In Eastern Mediterranean region, the WHO estimates the prevalence of anemia to be 32.4% in nonpregnant and 44.2% in pregnant women [2]. Anemia during pregnancy is a significant concern. During pregnancy, the fetal demand for iron increases maternal daily iron requirements around 10-fold, increasing from 6 mg/day to 22 mg/day in first and third trimesters of pregnancy, respectively. This increased demand for iron is covered mostly from maternal iron stores, which makes pregnant women at higher risk of developing iron deficiency and IDA [6].

The consequences of IDA during pregnancy are often serious and long lasting for both the mother and fetus. Mothers with anemia often experience increased fatigue levels, reduced exercise performance, and reduced mental performance [7]. Furthermore, severe anemia (Hb < 90 g/L) is related to an increased risk of premature delivery with subsequent low birth weight, small for gestational-age babies, and spontaneous abortion [6–10]. Additionally, maternal IDA may contribute to low iron status and poor health of infants. Also, pregnant women with anemia are at a greater risk of perinatal mortality and morbidity [10, 11]. Fetal iron metabolism is completely dependent upon maternal iron delivery via the placenta and so the effects of anemia on the fetus are directly related to the extent of maternal iron deficiency with increased mortality linked to severe IDA [6].

The prevalence of IDA varies among countries but is a major public health problem in the developing world, reflecting differences in race, socioeconomic factors, nutritional habits, medical care, and the frequency of parasitic illnesses [7].

In Palestine, the Ministry of Health (MoH) has protocols for IDA management and prevention that involves free iron supplements for pregnant women. In 2016, the overall prevalence of anemia among pregnant women (Hb <110 g/L) who visited maternal and child healthcare (MCH) centers of MoH was 29.5% (18.7% in West Bank region and 40.3% in Gaza Strip) [12]. Earlier in 2009 Khader et al. [13] reported an overall prevalence of anemia of 38.6% among pregnant women attending/accessing the UNRWA (United Nations Relief and Works Agency) clinics in Palestine with a substantial difference between those living in West Bank (31.1%) and Gaza Strip (44.9%). In the latter report [13], anemia prevalence revealed a significant increase in Gaza strip in 2009 (44.9%) compared to an earlier survey (35.7%) conducted in 2004 by UNRWA. The former report [12] surveyed the general population in Palestine excluding refugees who were surveyed by UNRWA [13]. Although these data revealed a high prevalence of anemia among pregnant women in Palestine, the association of anemia among Palestinian pregnant women with pregnancy outcome was not investigated. Therefore, this study aimed to assess the prevalence of anemia and IDA among pregnant women in Hebron Governorate (south of Palestine) and to investigate the association between maternal anemia and pregnancy outcome.

## 2. Materials and Methods

### 2.1. Study Subjects and Design

The study included 11 maternal and child healthcare (MCHC) centers distributed all over Hebron Governorate, Palestine. All pregnant women in their first trimester and presenting for the first time during current pregnancy at one of the MCHC centers covered in this study were asked to participate in this study. All participants were presenting at the MCHC centers for general medical examination related to the current pregnancy in the period March to October 2015. Pregnant women included in this study were contacted immediately after delivery (6-9 months after time of enrollment of pregnant women) and asked to include their newborns in the study. Inclusion criteria included apparently healthy according to the physical examination and laboratory data obtained at their first (current) visit, being not on iron supplementation, no previous pregnancy complications, and free of pregravid chronic diseases. A total of 300 pregnant women and 163 newborns born to them were included in the study.

All study subjects were briefed about the study and gave a written informed consent for themselves and on behalf of their babies. The principles of Helsinki Declaration were applied.

The Hebron Governorate (study region) is located in the southern part of West Bank and has a population of 0.729 million from around 3 million in West Bank, Palestine [14].

### 2.2. Data Collection and Analysis of Blood Samples

Anthropometric and socioeconomic data from the subjects were collected using a structured questionnaire. All pregnant women at their first trimester who met the inclusion criteria were asked to donate a blood sample and provide all information required to fill up the first part of the questionnaire concerned with the mothers. Collection of blood samples from pregnant women took place during their first visit to collaborating centers for the purpose of medical examination concerning current pregnancy. Participants were contacted immediately after delivery and asked for a permission to collect a blood sample from their newborns and provide all information needed to fill the second part concerned with the newborns. Blood samples were collected from newborn babies on the 5^th^ to 7^th^ day following delivery when babies were presenting at the MCHC center for their first vaccination date.

Blood samples were used for testing of Complete Blood Count (CBC) using Medonic (version CA 620; Boule Medical, Sweden) or Drew (version 6.3 model 2902-0531; Drew Scientific group, USA) hematology analyzers immediately after sample collection. Hematology analyzers were calibrated using the appropriate calibrators. Reliability of tests was assessed by running appropriate controls every day. Anemia was determined by hemoglobin concentration level less than 110 g/L for pregnant women based on the recommendations of WHO [15]. The severity of anemia was assessed based on WHO recommendations as follows: severe for Hb < 70 g/L, moderate for Hb 70-99 g/L, and mild for Hb 100-109 g/L [15].

Serum from mothers was collected, stored at -18°C, and used later for analysis of serum ferritin. Serum ferritin was determined using ferritin reagent kits and the automated chemistry analyzer machine (Abbott, USA) per the instructions of the manufacturer. For calibration, six ferritin standards (corresponding to ferritin levels of 0, 10, 50, 250, 500, and 1000 ng/ml) were used per the instructions of manufacturer. The ferritin standard calibrators were traceable to the WHO standards. Additionally, the ferritin controls (low, medium, and high) were assayed in each run for verification of the accuracy and precision of the test.

### 2.3. Statistical Analysis

Descriptive statistics including (mean and standard deviation) were calculated for all variables using SPSS software (Version 23). Inferential statistics were used to reach conclusions and make generalizations about the characteristics of populations based on data collected from the study subjects. These statistical tests include independent sample* t*-test, one-way ANOVA, and Chi-Square tests. A* P*-value < 0.05 was considered statistically significant.

## 3. Results

The general characteristics of pregnant women are described in Table 1. The average serum ferritin and Hb levels were within the normal range. However, out of 300 pregnant women, 77 women (25.7%) had Hb levels below 110 g/L and thus were considered anemic based on the recommendation of WHO [15]. Of the anemic pregnant women, 51 (17%) had mild anemia, 26 (8.7%) had moderate anemia, and none of the women had severe anemia, based on recommendation of WHO for assessment of anemia severity [15]. Analysis of serum ferritin levels showed that 156 pregnant women (52%) had serum ferritin levels below 15 ng/mL which indicates depleted iron stores (iron deficiency) based on the recommendations of the WHO [16]. Seventy-seven of the pregnant women (25.7%) were found to meet the clinical criteria (Hb < 110 g/L and serum ferritin < 15 ng/mL). These results indicate that all cases of anemia observed among our study subjects were due to iron deficiency. For the newborns, their average weight, gestational age, and Hb level were within the normal reference range (Table 2).

In order to examine the association between maternal characteristics and pregnancy outcome on one side with the maternal Hb levels on the other side, the mothers were divided into three Hb tertile groups (Table 3). A statistically significant difference was observed among the Hb tertile groups when compared with maternal serum ferritin, where serum ferritin levels increased with increasing Hb levels (Table 3). For the pregnancy outcome (newborns) a statistically significant difference was observed among maternal Hb tertile groups when compared with birth weight, birth height, and head circumference of newborns as well as with gestational age (Table 3). The results showed that birth weight, height, and head circumference of newborns as well as gestational age was increasing with increased maternal Hb levels.

As shown in Table 4, a statistically significant difference was observed between maternal serum ferritin levels when compared with maternal BMI kg/m^2^. For the pregnancy outcome, a significant difference between maternal serum ferritin levels was observed when compared with birth height and head circumference of newborns as well as with gestational age, where birth height, head circumference, and gestational age were lower in mothers with iron deficiency (serum ferritin <15 ng/mL) compared to mothers with normal serum ferritin (serum ferritin ≥ 15 ng/mL) (Table 4). The latter findings confirm the significant differences among Hb tertile groups when compared with these parameters (Table 3). Additionally, a statistically significant difference was observed between maternal serum ferritin levels when compared with frequency of low birth weight and frequency of preterm delivery (Table 4).

The correlation between maternal iron status and anthropometric indices versus pregnancy outcome was assessed by Pearson's correlation (Table 5). A significant positive correlation was observed between maternal Hb levels and newborns' birth weight, birth height, head circumference and gestational age. Similarly, a significant positive correlation was observed between maternal Hematocrit (Hct) levels and newborns' birth weight, birth height, gestational age, and Hb levels. Also, a significant positive correlation was observed between maternal serum ferritin levels and newborns' birth height, gestational age, and Hb levels. No significant correlation was found between maternal age, BMI, number of children, number of abortions, educational level, and monthly income on one side and pregnancy outcome on the other side (Table 5).

## 4. Discussion

The prevalence of IDA among pregnant women from Hebron Governorate was 25.7% and 52% of them had depleted iron stores (iron deficiency). These data indicate that anemia among pregnant in Hebron region is a public health problem that can be classified as moderate [15]. The prevalence of anemia among pregnant women reported in this study was within the average rates reported for several Arab countries [17–21] concerning pregnant women in their first trimester. In Jordan, Al-Mehaisen et al. [19] reported an overall prevalence of anemia of 34.7% among pregnant women in rural areas, and the prevalence rate ranged from 18.9% among women in first trimester to 32.7% in second trimester to 42.5% in third trimester. In Kuwait, the prevalence of anemia among pregnant women was reported at 24.1% [20]. In the latter report [20] the prevalence of anemia varied with the stage of pregnancy, being lowest in first trimester (14.8%), highest in second trimester (49.2%), and intermediate in third trimester (36%). In Saudi Arabia, the prevalence of anemia among pregnant women in their first trimester was reported at 29.6% [18] and 27.7% [21] in two different regions of Saudi Arabia. In our study, the percentage of pregnant women with depleted iron stores (52%) is higher than those with anemia (25.7%) in first trimester and this may indicate that the prevalence of anemia would become more common and more severe in second and third trimester.

Anemia or iron deficiency during pregnancy is associated with intrauterine growth retardation, premature birth, low birth weight, increased labor time, higher risk of infection, elevated maternal and prenatal mortality, muscle dysfunction, and low physical capacity [8, 9, 22–24]. In accordance with previous reports [9–11], our study revealed a significant difference among maternal Hb tertile groups when compared to newborns' birth weight, height, and head circumference as well as gestational age. The birth weight, height, and gestational age were lowest with maternal HbT1 (< 110 g/L) and highest with maternal HbT3 group (> 120 g/L). Although HbT1 (in our study included Hb values from 73 to 109 g/L) was not as low as Hb < 70 g/L, which is considered as severe anemia [15] the adverse effect of moderate/mild anemia on pregnancy outcome was clearly demonstrated. Additionally, there was a significant association between maternal low serum ferritin and frequency of low birth weight and frequency of preterm delivery.

The birth weight is affected by a complex and independent factors in addition to maternal Hb and serum ferritin. The anthropometry of the mother and her nutritional intake are thought to be among the most important [25, 26]. Among the anthropometry measures, prepregnancy BMI has received especial interest and was addressed by many studies. Despite this, the direct relationship between prepregnancy BMI and fetal growth is still not known [27]. Several reports have shown that underweight women or those with low BMI are at high risk of having low birth babies and other adverse pregnancy outcome [27–30]. Also, high BMI (but still in the normal category) has a favorable effect on pregnancy outcome, a BMI in the overweight/obese/severely obese categories has been reported to increase the risk of adverse obstetric and neonatal outcomes such as maternal diabetes, preterm delivery, macrosomia, increasing risk of admission of baby to intensive care unit, and perinatal death [30–32]. In the present study no significant correlation was observed between maternal anthropometric measures (age and BMI) and pregnancy outcome. Also, no statistically significant difference was observed among maternal Hb tertile groups when compared with maternal age and BMI; the maternal Hb showed a slight increase with increasing BMI (Table 3). Our findings concerning BMI and pregnancy outcome are contradictory to previous reports [30–32] and may be due to the limited number of overweight/obese women among the study subjects.

Furthermore, several studies have investigated the effect of socioeconomic status of the mother on pregnancy outcome. Anemia has been linked to poverty [1, 24, 33], which in turn is considered as a consequence of many factors including low monthly income and low levels of education. Therefore, in this study we have examined the association of the mother's socioeconomic status (monthly income, level of education, and parity) with maternal Hb, maternal serum ferritin, and low birth weight and did not find a significant correlation. In consistence with our findings, a previous study that investigated a cohort of pregnant women from Nablus-Palestine did not find a significant association between monthly income, level of education, and size of family and prevalence of anemia among this group [34]. While Khader et al. [13] reported a significant association between parity and prevalence of anemia among Palestinian pregnant women living in Refugee camps in West Bank and Gaza but no significant association was found with regard to level of education. A recent report from rural areas of Jordan has found no significant association between socioeconomic status of mothers and prevalence of anemia among pregnant women [19]. In contrast to our findings, a study from Malaysia found that older age of the mother, parity of four or above, Indian origin, and per capita monthly income are significant risk factors for low birth weight [27]. Another study from Nigeria found that less educated pregnant women had significantly higher prevalence of anemia compared to educated women, and no significant association between parity and prevalence of anemia [35]. Thus, further studies are needed to study the effect of the socioeconomic status of mothers' on the pregnancy outcome using a larger sample size and preferably from different regions in Palestine. Additionally, the role of some factors in the contradictory results concerning anthropometric parameters among different studies from different geographic regions such as prevalence rate of anemia, ethnicity, and local environmental factors may be better tackled by meta-analysis studies.

A limitation in our study is that only one blood sample was collected from pregnant women and that was in their first trimester. However, anemia usually becomes more common and more severe as pregnancy progress. Thus, a second blood sample collected at the end of second trimester or beginning of third trimester could provide more information on the association between maternal anemia in second or third trimester and pregnancy outcome compared to first trimester.

## 5. Conclusion

Our study indicated that IDA is a moderate public health problem among pregnant women in Hebron Governorate and more than half of study subjects have depleted iron stores. Maternal Hb and serum ferritin were found to affect pregnancy outcome (birth weight, height, and gestational age). Newborns born to women with low Hb levels tended to have lower birth weight and height, head circumference, and lower gestational age. No significant association was observed between maternal anthropometric measures (age and BMI) or the socioeconomic status (level of education, monthly income, and parity) and pregnancy outcome. The high prevalence of anemia in our subjects was probably due to low iron intake and poor dietary habits rather than food insecurity or disease. Therefore, the etiological factors associated with maternal anemia during pregnancy in Palestine should deserve more attention.

## Figures and Tables

**Table 1:** General characteristics of the study subjects/pregnant women at first trimester.

**General characteristics**					
	n	Mean ± SD	95% CI	Minimum value	Maximum value
Age (years)	300	26.3 ± 5.9	25.6 – 26.9	15	45
BMI (kg/m^2^)	300	24.8 ± 4.1	24.3 – 25.2	15.2	41.2
No. of children	300	2.1 ± 2.0	1.9 – 2.4	0	10
No. of abortions	300	0.66 ± 1.18	0.53 – 0.79	0	8
Educational level (years)	300	11.6 ± 3.2	11.3 – 12.0	0	16
Monthly income (NIS)*∗*	264	1832 +-1154	1700 - 1963	700	7000
**Biochemical status**					
Hb (g/L)	300	118 ± 14	117 – 120	73	153
Hct (%)	300	34.1 ± 3.7	33.7 – 34.5	24.5	49.3
Serum ferritin (ng/mL)	300	20.2 ± 23.2	17.5 – 22.8	1.1	192.0

*∗*NIS: New Israeli Sheqel, the currency used mostly in Palestine.

**Table 2:** General characteristics of the newborns (pregnancy outcome).

**General characteristics**					
	n	Mean ± SD	95% CI	Minimum value	Maximum value
Birth weight (gm)	163	3022 ± 585	2932 - 3113	1900	4350
Birth height (cm)	96	48.3 ± 2.7	47.8 – 48.9	43	55
Head circumference (cm)	96	34.6 ± 1.4	34.3 – 34.9	31	37
Gestational age (weeks)	122	38.5 ± 1.5	38.5 – 38.7	33	40

**Biochemical status**					

Hb (g/L)	79	162 ± 22	157 – 167	111	214

**Table 3:** **Maternal characteristics and pregnancy outcome by Hb tertile groups. **Statistical analysis was performed by one-way ANOVA, except for frequency of low birth weight and frequency of preterm delivery that were analyzed by Kruskal-Wallis test.

	**All**	**HbTertile groups**			
	**(Mean ± SD)**	**HbT1**	**HbT2**	**HbT3**	***P-*value**
		**Hb< 110 g/L**	**Hb 110- 120 g/L**	**Hb> 120 g/L**	
**Maternal characteristics **(n= 300)*∗*					

Serum ferritin (ng/mL)	20.2 ± 23.2	14.8 ± 30.2	15.9 ± 10.6	25.0 ± 22.6	0.001
Age (years)	26.3 ± 5.9	27.1 ± 6.2	25.8 ± 6.3	26.1 ± 5.4	0.324
BMI (kg/m^2^)	24.8 ± 4.1	24.3 ± 3.2	24.4 ± 4.5	25.1 ± 4.2	0.250
No. of children	2.1 ± 2.0	2.4 ± 2.1	2.2 ± 2.2	2.0 ± 1.9	0.268
No. of abortions	0.66 ± 1.2	0.73 ± 1.03	0.52 ± 0.94	0.69 ± 1.35	0.503
Educational level (years)	11.6 ± 3.2	11.9 ± 3.5	11.4 ± 3.0	11.6 ± 3.1	0.657
Monthly income (NIS)	1832 ± 1154	1888 ± 1262	1667 ± 913	1883 ± 1201	0.375

**Pregnancy outcome **					

Birth weight (gm)	3022 ± 585	2817 ± 568	3030 ± 577	3144 ± 571	0.009
	(n= 163)^1^				
Birth height (cm)	48.3 ± 2.7	47.3 ± 2.5	48.3 ± 2.5	49.1 ±2.8	0.022
	(n= 96)^1^				
Head circumference (cm)	34.6 ± 1.4	34.1 ± 1.5	34.5 ± 1.2	35.0 ± 1.3	0.017
	(n= 96)^1^				
Gestational age (weeks)	38.5 ± 1.5	37.8 ± 1.5	38.6 ± 1.4	38.8 ± 1.6	0.012
	(n= 122)^1^				

	**Frequency (%)**	**HbT1**	**HbT2**	**HbT3**	***P-*value**

Frequency of low birth weight (< 2500 gm)	39	16	9	14	0.053
	(23.9%)	(9.8%)	(5.5%)	(8.6%)	
Frequency of preterm delivery (< 37 weeks)	16	8	4	4	0.53
	(13.1%)	(6.6%)	(3.3%)	( 3.3%)	

*∗*n= 300 for all maternal characteristics except for monthly income it was 264. ^1^The indicated numbers of samples were assessed for each parameter.

**Table 4:** **Maternal characteristics and pregnancy outcomes by maternal serum ferritin levels. **Statistical analysis was performed by independent sample *t*-test, except for frequency of low birth weight and frequency of preterm delivery that were analyzed by Chi-square test.

	**All ** **(Mean ± SD)**	**Serum ferritin**		
		**Normal**	**Iron deficiency**	***P-*value**
		**(**≥ **15 ng/mL)**	**(< 15 ng/mL)**	
**Maternal characteristics **(n= 300)*∗*				

Age (years)	26.3 ± 5.9	26.0 ± 5.4	226.3 ± 6.3	0.436
BMI (kg/m^2^)	24.8 ± 4.1	25.3 ± 4.4	24.2 ± 3.7	0.017
No. of children	2.1 ± 2.0	2.0 ± 1.9	2.3 ± 3.1	0.158
No. of abortions	0.66 ± 1.2	0.59 ± 1.1	0.72 ± 1.2	0.328
Educational level (years)	11.6 ± 3.2	11.8 ± 2.8	11.5 ± 3.5	0.439
Monthly income (NIS)	1832 ± 1154	1835 ± 1096	1829 ± 1209	0.961

**Pregnancy outcome **				

Birth weight (gm)	3022 ± 585	3123 ± 538	2945 ± 610	0.053
	(n= 163)^1^			
Birth height (cm)	48.3 ± 2.7	49.1 ± 2.3	47.9 ± 2.8	0.026
	(n= 96)^1^			
Head circumference (cm)	34.6 ± 1.4	35.0 ± 1.1	34.4 ± 1.4	0.025
	(n= 96)^1^			
Gestational age (weeks)	38.5 ± 1.5	38.9 ± 1.5	38.1 ± 1.5	0.008
	(n= 122)^1^			

	**Frequency**	**Normal**	**Iron deficiency**	***P-*value**
		**(**≥ **15 ng/mL)**	**(< 15 ng/mL)**	

Frequency of low birth weight (< 2500 gm)	39 (23.9%)	9 (5.5%)	30 (18.4%)	0.001
Frequency of preterm delivery (< 37 weeks)	16 (13.1%)	2 (1.6%)	14 (11.5%)	0.003

*∗*n= 300 for all maternal characteristics except for monthly income it was 264.

^1^The indicated numbers of samples were assessed for each parameter.

**Table 5:** Pearson's correlation coefficients between pregnancy outcome (newborns) and maternal iron status and anthropometric/socio-economic factors.

**Maternal data↓**	**Newborn data**				
	Birth weight	Birth height	Head circumference	Gestational age	Hb
Hb	**0.191** **∗**	**0.307** **∗** **∗**	**0.257** **∗**	**0.275** **∗** **∗**	0.197
Hct	**0.171** **∗**	**0.224** **∗**	0.178	**0.228** **∗**	**0.235** **∗**
Serum ferritin	0.008	**0.216** **∗**	0.174	**0.254** **∗** **∗**	**0.287** **∗** **∗**
Age	0.025	0.103	0.124	0.038	0.052
BMI	0.061	0.074	0.022	-0.016	0.008
No. of Children	0.126	0.107	0.130	0.147	-0.052
No. of abortions	0.046	0.054	0.088	-0.037	0.001
Educational level	-0.023	-0.138	-0.080	-0.062	0.170
Monthly income	0.095	-0.033	-0.020	0.167	0.106

*∗* Correlation is significant at the 0.05 level (2-tailed). *∗∗* Correlation is significant at the 0.01 level (2-tailed).

## Data Availability

The raw data (CBC and anthropometric data) used to support the findings of this study are available from the corresponding author upon request.
